# Design and development of electrostatic brakes on the filament level

**DOI:** 10.1038/s41598-025-86243-0

**Published:** 2025-02-13

**Authors:** Philippa R. C. Böhnke, Nadja Schenk, Carola Böhmer, Hans Winger, Iris Kruppke, Andreas Nocke, Johannes Mersch, Ercan Altinsoy, Chokri Cherif

**Affiliations:** 1 Centre for Tactile Internet with Human-in-the-Loop (CeTI), Technische Universität Dresden, 01069 Dresden,, Germany; 2 Institute of Textile Machinery and High Performance Material Technology, Technische Universität Dresden, 01069 Dresden, Germany; 3 Chair of Acoustics and Haptics, Institute of Acoustics and Speech Communication, Technische Universität Dresden, 01069 Dresden, Germany

**Keywords:** Electrostatic brake, Capacitor, Electrically conductive, Yarn, Theoretical concepts, Passive actuator, Proof-of concept, Electrical and electronic engineering, Mechanical engineering

## Abstract

The Cluster of Excellence ‘Centre for Tactile Internet with Human-in-the-Loop (CeTI)’^1^ addresses developments and inventions for the use in or as smart devices in many areas, such as Industry 4.0, medicine and skill learning. The application of sensor units in smart textiles is widespread and used in various industry branches. Besides sensors, the development of textile actuating units is a relevant research topic. This paper discusses a theoretical actuator concept that leads to a ready-to-implement fiber-based electrostatic brake concept (passive actuator). Generally, the set-up is similar to a capacitor. Two different variants are presented according to the design of the dielectric and outer electrode layer. The dielectric material, its thickness, manufacturing process, future properties and implementation possibilities of the concepts are considered. Finally, a proof of concept with first results is presented.

## Introduction

The global wearable market is growing rapidly^1–4^, and smart textiles are part of it. They are defined as ‘textiles that can sense and/or respond to their environmental conditions or stimuli’^5^. These stimuli can be mechanical, thermal, magnetic, chemical, electrical or from other sources^4–6^. The application of smart textiles can be found in many fields, including healthcare, industry and wearable electronics^2,3,7^. Smart textiles can be divided into passive, active and smart wearables^4,5^. Passive smart textiles have additional functions, such as anti-bacterial, anti-static or anti-odour properties^5^. Active smart textiles can sense and respond to stimuli from the environment and react to them^5^. This category includes textile sensors and actuators. Highly intelligent textiles sense stimuli, react to them and adapt to the conditions^8^.

This paper deals with the second group, active smart textiles. In particular, an actuating concept based on electrostatic forces, namely an electrostatic brake (EB), is discussed and developed. An EB is structured like a capacitor, but with a sliding dielectric layer. Its use as a passive actuator offers many opportunities to fulfil actuator tasks, especially in combination with virtual reality environments, e.g. braking movements to give the feeling of grasping something. Downscaling from the macroscopic to fiber-scale opens up new areas for integration processes such as textile processing techniques. Although the manufacturing process and weight of the macroscopic scale EB are very advantageous, there are many possibilities when downscaled to the filament or yarn level. Firstly, the actuator could be integrated directly into the production process of smart clothing, for example by knitting or weaving. Moreover, less material is used, the same capacitance can be achieved with a smaller system, and the product becomes more user-friendly as it is enclosed and less prone to damage. In order to implement a filament-based EB at a later stage, requirements need to be defined. The radii are based on yarns typically used in garments. Voltage and further parameters are orientated towards Hinchetet al.^9^ and additional literature.

Hinchetet al. developed an EB integrated into a glove. The principle is based on a capacitor: a thin insulating dielectric layer separates two conductive layers. When a voltage is applied between two electrodes, an electrostatic force is created between them, mechanically locking them together. In^9^ the capacitor itself consists of two stainless steel strips (180 mm long, with overlap length of 110 mm, 10 mm wide and 100 μm thick) and a thin insulating layer made of polyimide (PI, 13 μm thickness) bonded to one of the metal strips with a conductive adhesive. The following formula describes the capacitance obtained with this system, including the relative permittivity ε_0_ of vacuum and the dielectric layer (ε_r_), the surface A and thickness d of the dielectric layer:

1 $$\:{C}_{\:}=\:\frac{{\epsilon\:}_{r}\bullet\:\:{\epsilon\:}_{0}\:\bullet\:A}{d}$$

In addition, the compressive force is calculated using the following formula with U as the voltage. Hinchetet al. applied a voltage of 1.5 kV to obtain a theoretical compression force of 220 N. If the compression force multiplied by the friction, coefficient is equal to the friction force or even bigger, the system is working, i.e. it will lock on demand. With the electrostatic brake after Hinchetet al., holding forces of up to 20 N can be achieved.

2 $$\:{F}_{compression}=\:\frac{{\epsilon\:}_{r}\:\bullet\:\:{\epsilon\:}_{0}\:\bullet\:A\:\bullet\:{U}^{2}}{{2\bullet\:d}^{2}}=C\:\bullet\:\:\frac{{U}^{2}}{2\:\bullet\:d}$$

**Fig. 1:**
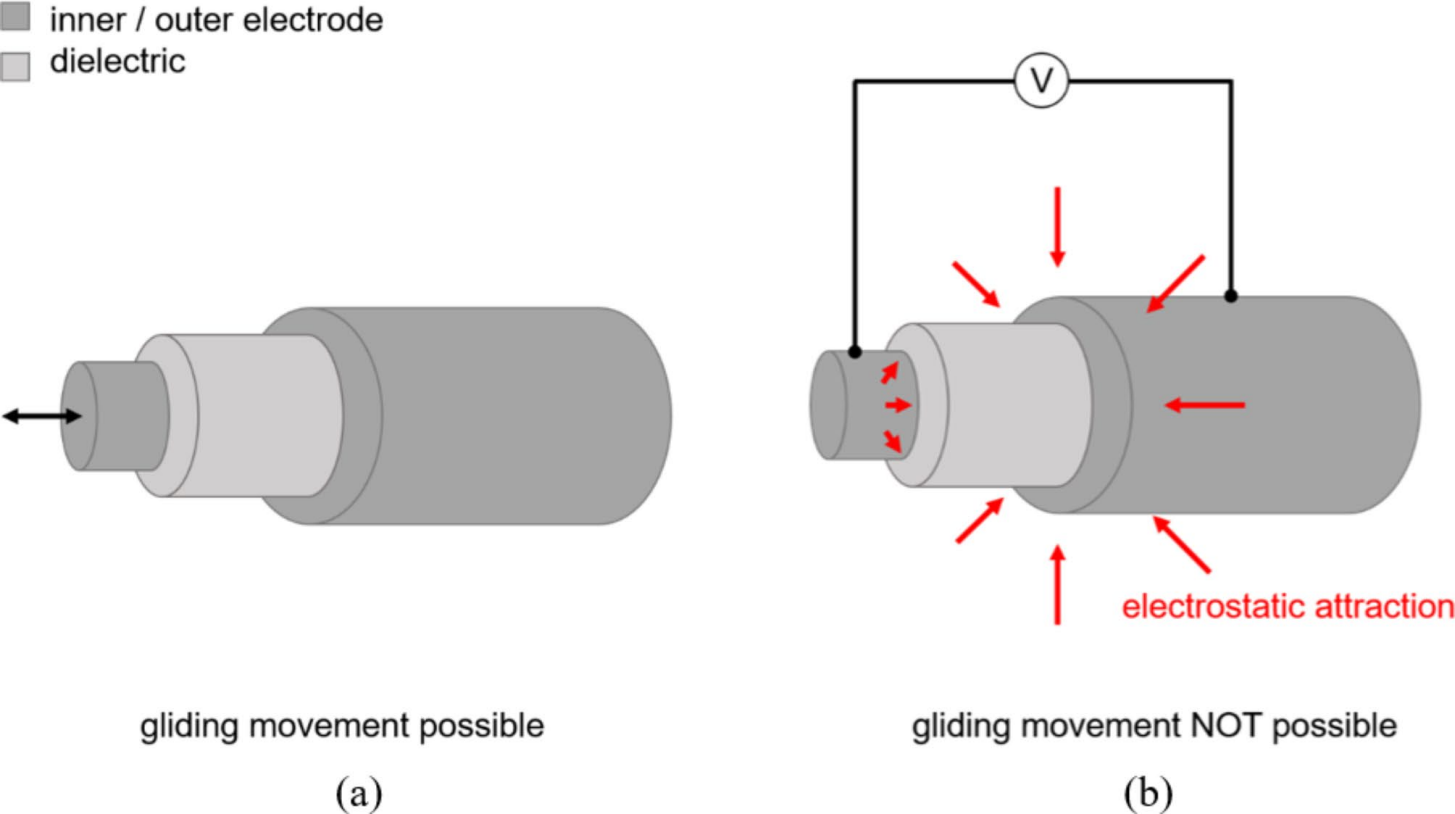
Schematic representation of the mode of operation based on a cylindrical structure with no voltage on (**a**) and voltage on (**b**).

As described above, two possible concepts are presented in this paper. Both concepts are based on cylindrical capacitors with an inner electrode, a dielectric layer and an outer electrode. Figure 1 shows with a schematic of the working principle based on a cylindrical structure. Figure 1(a) describes the possible sliding movement of the inner electrode and the dielectric layer when no voltage is applied. Figure 1(b) shows the effect of applying a voltage, resulting in the electrostatic attraction of the inner and outer electrode. Consequently, sliding of the inner electrode and dielectric layer is no longer possible.

**Fig. 2:**
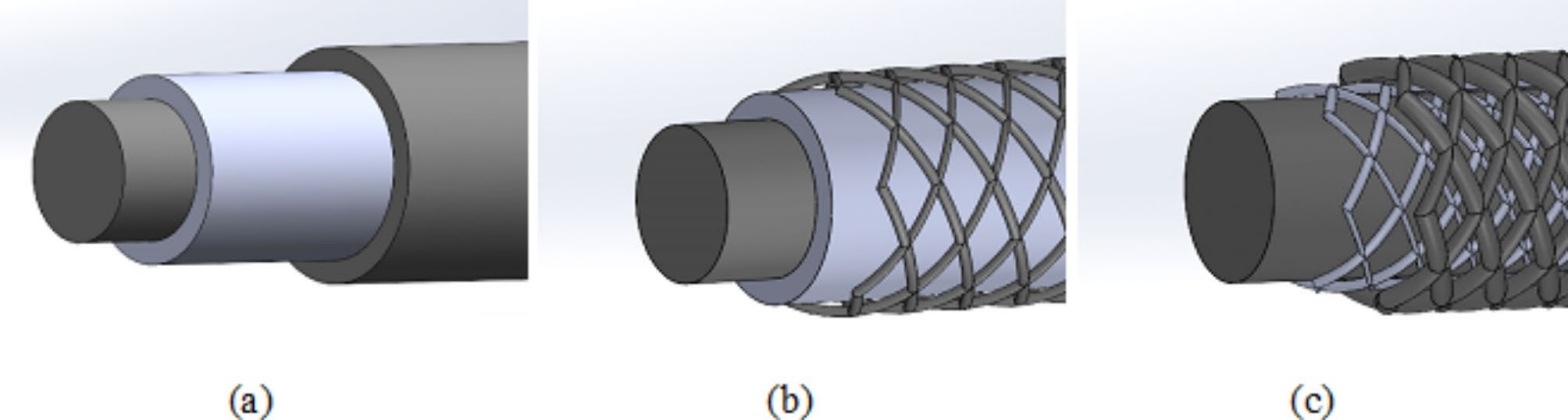
Set-up of closed system (CS) (**a**), semi-open system (SeOS) (**b**) and open system (**c**) (OS) of the possible concepts of electrostatic brake.

Three different actuator systems for fiber applications are shown in Fig. 2.

On the one hand, the closed system (CS) shown in Fig. 2(a) consists of an inner electrode coated with a dielectric layer and an outer electrode. The capacitance can be calculated using the following formula; where l is the length and r is the outer diameter.

3 $$\:{C}_{CS}=\:\frac{2\:\bullet\:\:\pi\:\:\bullet\:\:{\epsilon\:}_{r}\:\bullet\:\:{\epsilon\:}_{0}\:\bullet\:l}{{log}\left(\frac{r}{r-d}\right)}$$

On the other hand, the **semi-open system (SeOS)** shown in Fig. 2(b) consists of an inner electrode with a dielectric layer and an outer electrode layer consisting of six braids. The system shown in Fig. 2(c) consists of an inner electrode with a braided or wound dielectric and an outer electrode layer (consisting of 6 braids), calls the **open system (OS)**. The capacitance of both systems (SeOS and OS) is calculated according to the Lecher conductor formular.

4 $$\:{C}_{OS\_SeOS}=\:\frac{6\:\bullet\:\:\pi\:\:\bullet\:\:{\epsilon\:}_{r}\:\bullet\:\:{\epsilon\:}_{0}\:\bullet\:\: l}{arcosh(1+\frac{d}{2\:\bullet\:r})}$$

The following literature represents the current state of cylindrical capacitors. FRUTIGER et al.^10^ developed a multi-core shell fibre consisting of four concentric layers with an ionically conductive liquid core, surrounded by a layer of dielectric elastomer. One layer of the liquid is then encapsulated by a layer of silicone. This capacitor was produced by a printing process and was mainly for sensing rather than actuation. Dip coating has been used to produce cylindrical capacitors as demonstrated by Kofodet al.^11^. , where a multilayer coaxial dielectric elastomer actuator was produced by repeatedly dipping a rubber fiber in conductive and dielectric precursors. Wanget al.^12^ developed thin capacitive strain sensors by melt spinning with a styrole-ethylene-butylene-styrole (SEBS) core filament and wrapping multi-walled carbon nanotube aerogel sheets onto the pre-stretched (900%) SEBS core. Probstet al.^13^ developed a bicomponent EC multifilament consisting of thermoplastic polyurethane (TPU) mixed with carbon nanotubes (CNT). The filament has a fineness of 2171 ± 422 tex and a resistivity of 110 ± 39 Ωcm^− 1^ at 0% elongation. Merschet al.^14^ further investigated CNT filled TPU with filler contents of 4 wt%, 5 wt% and 6 wt% using cyclic electromechanical testing. The investigations were complemented by the development of a conductive TPU coating that can be applied by dip coating^15^.

Capacitors can also be 3D printed. Blazet al. developed three types of 3D printed rolled capacitors based on conductive acrylnitril-butadien-styrol (ABS) composite electrodes. These capacitors reached a capacitance of 5119 pF at a frequency of 100 Hz. However, this is a rolled planar structure with a length of only 10 mm, which is not long enough for the development of a yarn-like actuator. Jaksicet al.^16^ presented a 3D printed capacitor made of electrically conductive filaments using fused filament fabrication, which achieved a capacitance of 62 pF. However, they did not develop a cylindrical structure.

Following the example of slippery coatings for fibers, Yinet al.^17^ developed a MXene-based composite coating via in-situ formed nanostructured tribofilm. The research resulted in a unique lubrication mechanism for the coating. However, the MXene-based composite coating is highly conductive^18^ and therefore not suitable for use as a dielectric. Khattabet al.^19^ investigated the development of a lanolin/silicone coating to provide a water repellent surface for viscose fibers. In addition to the goal of water repellency objective, lower friction is also noted. Silicone offers a good opportunity to produce a low friction coating for fibers. However, further development is needed to adapt the coating to the respective electrode substrate.

## Materials and methods

### Development of concepts

In order to be able to compare the concepts, their mathematical foundations ($\:{C}_{CS}$ for CS and $\:{C}_{OS\_SeOS}$ for OS and SeOS) have been computer modelled. Different theoretical methods are used to compare the two concepts described above. The computer-aided design (CAD) software Solidworks (Dassault Systèmes, Vélizy-Villacoublay, France) is used for the visualization of the concepts. In addition, Matlab (The MathWorks Inc., Natick, Massachusetts) is used to analyse the potential capacitance and the compressive force. The starting point of the analysis is the mathematical background of the systems derived in the formulae 3 and 4. The mathematical relationships are greatly simplified. The air gap between the electrodes necessary for the operation of the ES brake and its influence on the dielectric constant are not considered, only the different geometric shapes of the capacitors and their influences. The analysis focuses on the influence of different dielectric materials, the thickness of the dielectric layer, and the diameter of the inner electrode. Due to the large number of variables involved, two different analysis cases were defined. In the first case, the influence of the dielectric constant (Table 1) is examined, with a fixed inner diameter of 0.2 mm assumed. In the second case, the influence of the diameter size is investigated, with investigated and assumed dielectric constant of PU (4,3 F m^− 1^). In both cases, the models with an electrode length of 20 mm and an activation voltage of 500 V are considered.

**Table 1:** Selected relative permittivity of dielectric materials^20–23^.

Material	Abbreviation	Dielectric strength [kV/mm]	Relative permittivity [F/m]
Polyethylene	PE	40	2.3
Polypropylene	PP	200	2.4
Polyvinyl chloride	PVC	150	3.3
Polytetrafluoroethylene	PTFE	20	2.0
Polyvinylidene fluoride	PVDF	40	8.0
Polyamide	PA	15	3.5
Polyethylenterephthalat	PET	25	4.4
Acrylnitril-Butadien-Styrol	ABS	41	3.3
Polystyrole	PS	43	2.6
Silicone	SI	18	3.0
Thermoplastic polyurethane	TPU	39	8.2
Polyurethane	PU	18−14	4.3
Air	-	-	1.0

### Proof-of-concept

To demonstrate the viability of the OS and SeOS concepts, two different systems were developed, each using a different combination of materials and manufacturing techniques. PU-SeOS were fabricated to validate the SeOS concept. The Ba-OS was developed to validate the OS. The PU-SeOS included a conductive hybrid yarn consisting of two polyester (PET) fibres and a silver-coated PA fibre from AMANN & SÖHNE GMBH & CO. KG (Bönningheim, Germany) as the core, a polyurethan (PU) coating as the dielectric layer and braided copper wire as the outer electrode (**PU-SeOS**). The second system (**Ba-OS**) consists of the same conductive hybrid yarn as the core, a dielectric braid made of PET multifilament and a copper braid as the outer electrode. The variation braiding machine VF ¼-32-140 from Herzog GmbH (Oldenburg, Germany) is used to braid the layers of all systems with six braids. Table 2 shows the designation of the systems and the different braiding densities of the layers of each system.

**Table 2:** Braiding density of the layers of the EB system.

	System components	Braiding density of the braided dielectric layer	Braiding density of braided core electrode
Ba-OS	silver-plated yarn PET-Multifilament copper wire	5 braids/cm (PET-multifilament)	15 braids/cm (thick copper wire)
PU-SeOS	silver-plated yarn PU-coating copper wire	---	15 braids/cm (thick copper wire)

### Measuring method

Optical characterization of the systems was performed using a light microscope (AXIOImager.M1m from Carl Zeiss AG, Oberkochen, Germany) with incident light and bright field at magnifications ranging from 50x to 500x. In order to analyse the cross-section, the specimen is embedded in epoxy resin. Additionally, the capacitance of the two different variants was measured using a precision multimeter (Keithley DAQ 6510, Cleveland, Ohio) with a high-performance calculator at a sample length of 20 mm.

**Fig. 3:**
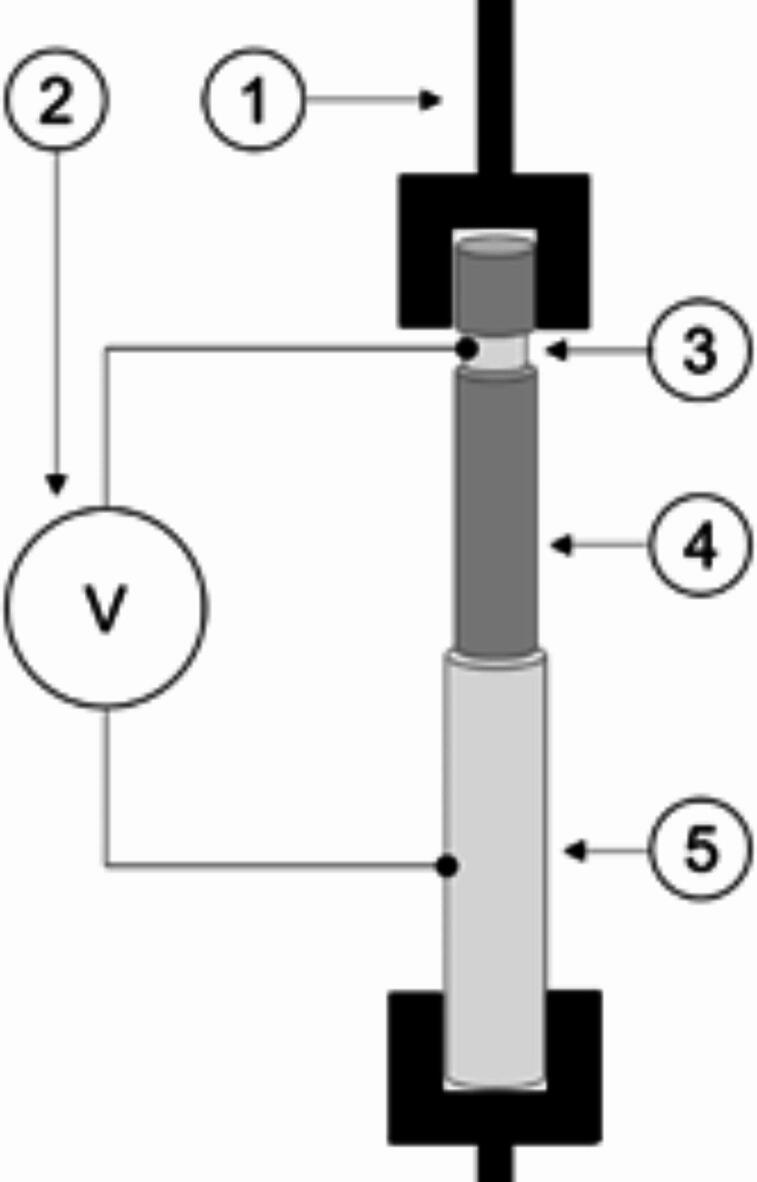
Schematic picture of electrical pullout test set-up.

A test rig has been developed to test the actuation performance of the systems, as shown in the Fig. 3. For this purpose, a tensile testing machine with a 5 N load cell (Zwick Zmart.Pro by ZwickRoell, Stadt Germany) is combined with a high voltage source (up to 5 kV) as shown in Fig. 3. A DC voltage of 500 V is applied. Before testing, the samples are prepared by cutting them to a length of 40 mm, then pulling the core out by hand for 20 mm (4) and removing the dielectric layer from the core (3). The specimen is then clamped (1) at the core (3) and the outer electrode (5) in the insulated tensile tester machine and connected to the high voltage source (2). During the test, the core and the dielectric layer of the PU-SeOS were pulled out due to the strong connection between both components resulting from the application of the coating on the core. In contrast, in the Ba-OS only the silver-plated yarn was pulled out. The test is performed at a speed of 4 mm/min over a pullout length of 10 mm. The force of the systems was quantified in two distinct states: one in which the voltage was absent (i.e., the voltage off state) and another in which the voltage was present (i.e., the voltage on state). Each system was tested in both the “voltage off” and “voltage on” using a sample amount of three. The results of the pull-out test without voltage were subtracted from the results of the pull-out test with voltage in order to observe the pure influence of the application of voltage on the pull-out force. For example, a force of 1.2 N was measured in the no voltage test and a force of 1.8 N was measured in the voltage on test, so the true pull-out force is 0.6 N when voltage is applied. This test rig measures the pull-out force. However, for validation purposes, the systems have a compression prediction for actuation performance. When voltage is applied, the system is compressed, resulting in an increase in compression force, making it more difficult to pull out the core. The increase in force is quantified as the pull-out force. This test rig therefore measures the compression force indirectly through the pull-out force.

## Results and discussion

### Analysis of the theoretical capacitance and compression force of the concepts

#### Influence on capacitance

In the following section, all concepts concerning the material of the dielectric layer, its thickness and the diameter of the inner electrode and their effect on the capacitance according to formulae (3) and (4) are analysed.

**Fig. 4:**
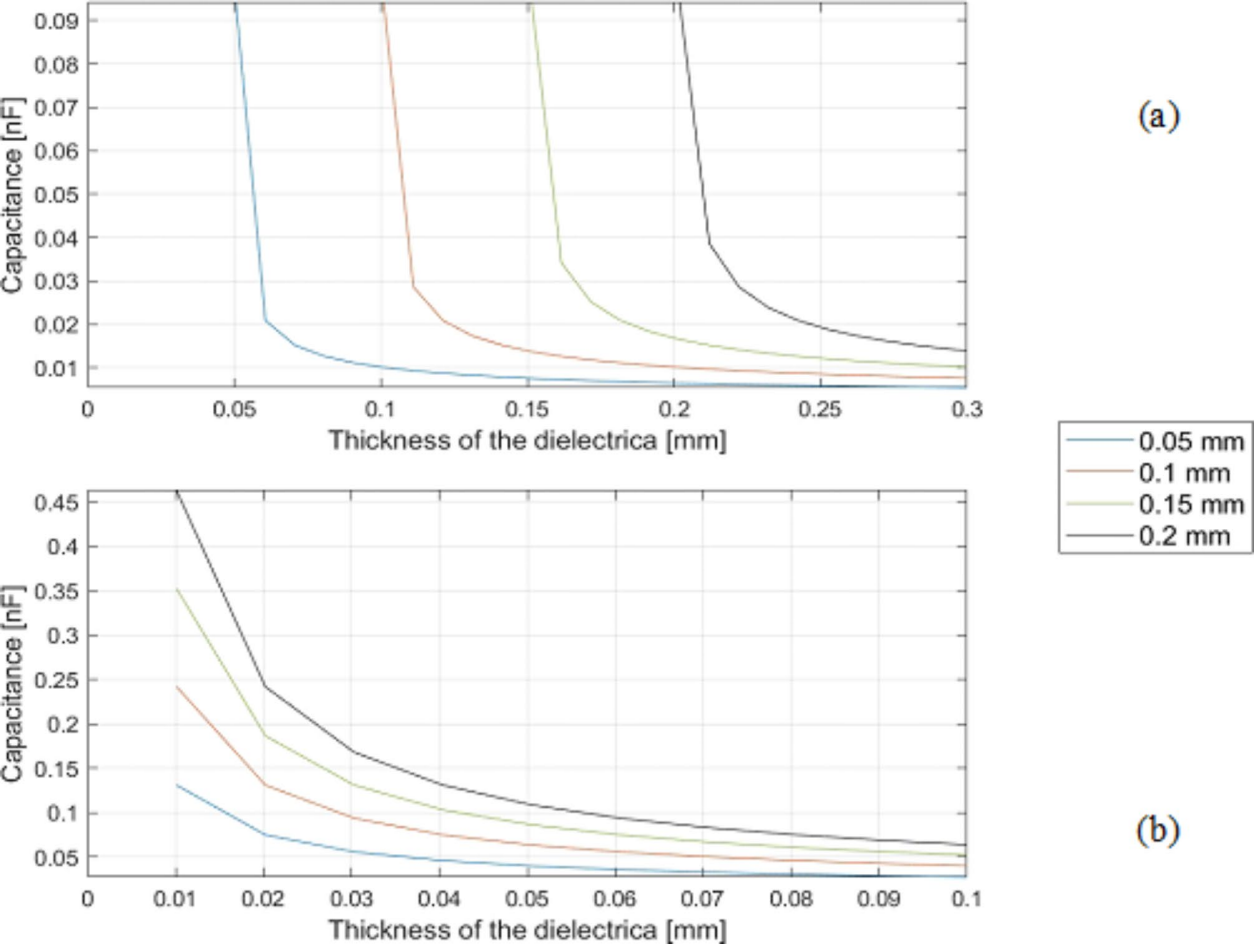
Influence of the diameter of the inner electrode on capacitance of (**a**) closed system (CS) and (**b**) semi-open/ open system (SeOS/OS).

Figure 4 shows the resulting capacitance of CS (a) and SeOS/OS (b) with different diameters of the inner electrode and increasing dielectric layer thickness up to 3 ∙ 10^− 4^ m. Here, the calculated capacitance values become larger as the inner diameter increases. In this system the values are calculated from a dielectric thickness of 0.01 mm, irrespective of the diameter of the inner electrode. In SeOS/OS the capacitance values remain the same regardless of the inner diameter. Capacitance values are calculated from a dielectric thickness of the same order of magnitude as the inner diameter. The calculated capacitance values of the CS are larger compared to the SeOS/OS.

**Fig. 5:**
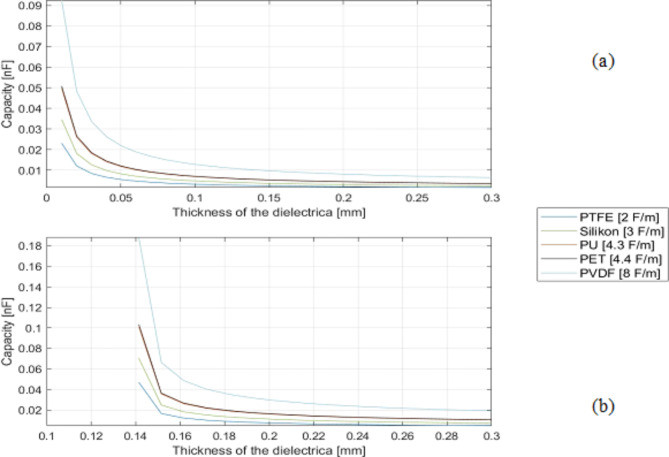
Influence of dielectric material and thickness on capacitance of (**a**) closed system (CS) and (**b**) semi-open/ open system (SeOS/OS).

Figure 5 shows the resulting capacitances of CS (a) and SeOS/OS (b) with different dielectric materials and increasing dielectric layer thickness up to 3 ∙ 10^− 4^ m. For CS it is clear that the thinner the layer thickness, the higher the capacitance. Both systems show that the higher the permittivity of the dielectric material, the higher the capacitance. (e.g., PVDF and TPU). The capacitance values of CS are higher than those of SeOS/OS. The calculation threshold for CS is also 0.1 ∙ 10^− 4^ m and for SeOS/OS values of 1.3 ∙ 10^− 4^ m are calculated for all graphs.

#### Influence on compression force

In the following section the compression force of both systems is calculated concerning the material of the dielectric layer, its thickness and the diameter of the inner electrode according to formula (2).

**Fig. 6:**
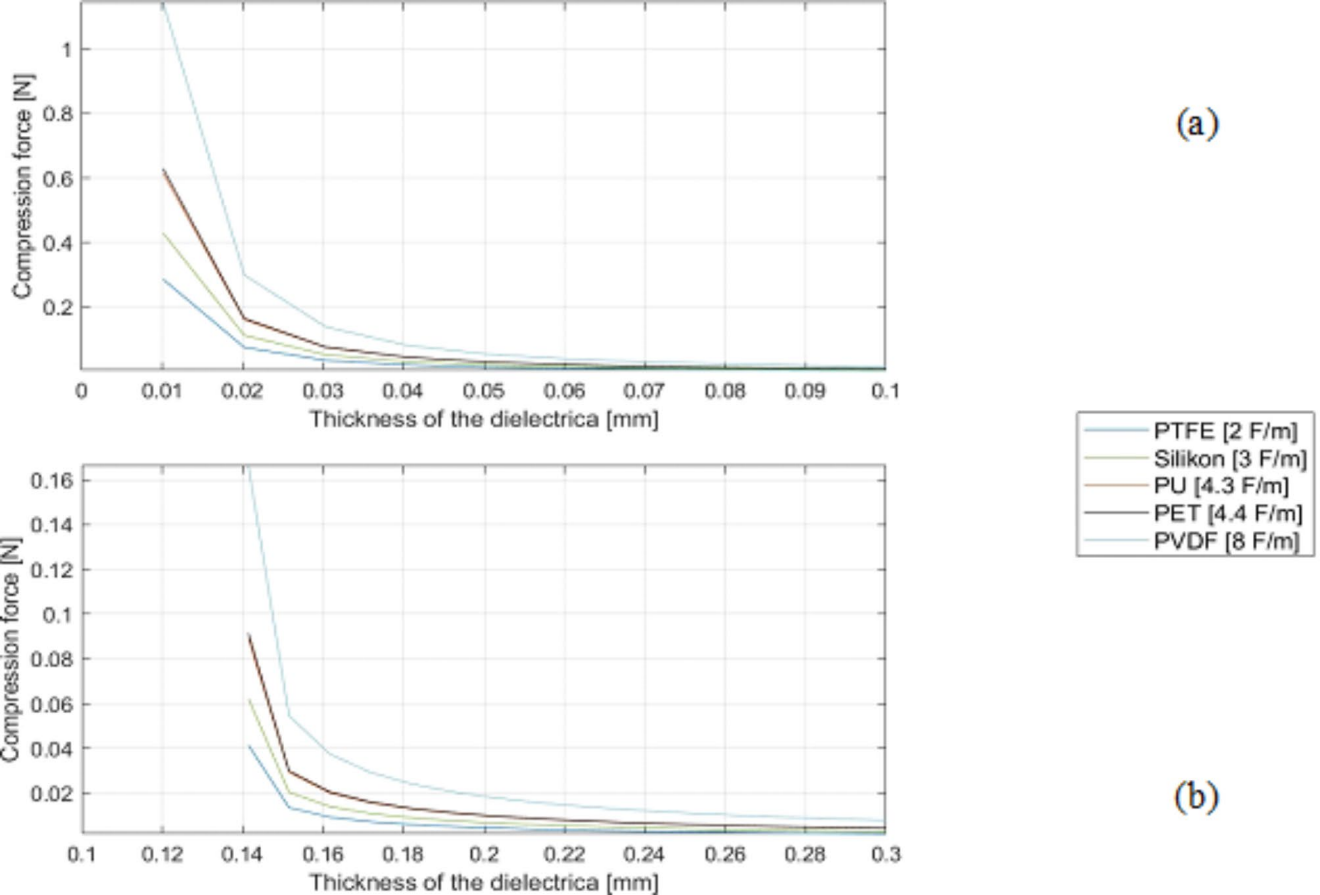
Influence of dielectric material and thickness on compression force of (**a**) closed system (CS) and (**b**) semi-open/ open system (SeOS/OS).

Figure 6 shows the resulting compression forces of CS (a) and SeOS/OS (b) with different dielectric materials and increasing thickness of the dielectric layer thickness up to 2 ∙ 10^− 4^ m. It also increases with thinner dielectric layer, regardless of the material of the dielectric layer material. However, the results of the SeOS/OS for the calculation of the compressive force and the capacitance are different. The compressive forces decrease with larger inner diameter. The calculation limits of the two systems are the same for the capacitance calculation.

**Fig. 7:**
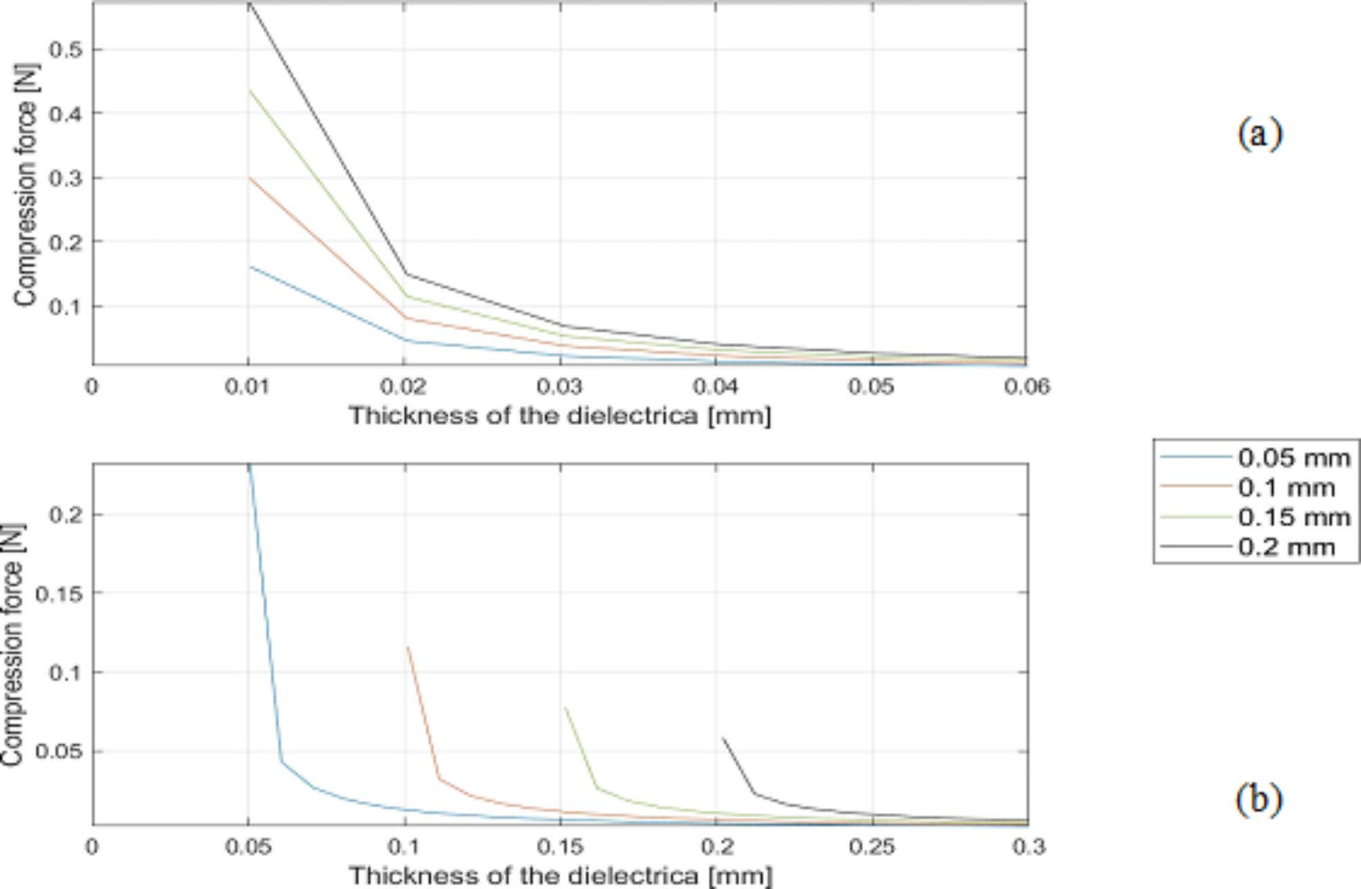
Influence of the diameter of the inner electrode on compression force of (**a**) closed system (CS) and of the (**b**) semi-open/ open system (SeOS/OS).

Figure 7 illustrates the resulting compression forces of CS (a) and SeOS/OS (b) with different dielectric materials and an increasing thickness of the dielectric layer up to 3 ∙ 10^− 4^ m. The general statements of the diagram are similar to those of the capacitance. It can be observed that the achievable forces of the CS are higher than those of the SeOS/OS.

A comparative analysis of the simulated investigations of the CS and Se-OS/OS capacitor designs reveals notable discrepancies in the magnitude of the compression forces and capacities. The CS exhibits higher values. In addition, in the SeOS/OS, values are obtained when the layer thickness is equal to or greater than the core diameter. In contrast, in the CS, values are determined for each core diameter from a layer thickness of 0.01 mm. In the SeOS/OS, the smallest core diameters give the highest compression and capacitance values, while the reverse is true for the CS. In essence, the highest values are attained in both capacitor systems when a relatively thin dielectric layer is present, the dielectric constant is high and the capacitor has a longer expansion.

### Implementation of concept

In order to implement these concepts, a number of material requirements must be satisfied. The core is discussed in terms of its essential requirements, namely that it should be electrically conductive, sufficiently stiff and non-hazardous^2^. Potential materials can be intrinsic electrically conductive polymers, extrinsic electrically conductive polymers^9^, carbon fibres, metallized polymers, metallic wires and numerous others. It is essential that the dielectric layer exhibits a high dielectric constant and a low coefficient of friction, while also demonstrating excellent adhesion to one of the electrodes^2^. The selection of an appropriate dielectric material is contingent upon the chosen manufacturing method. The CS allows for the implementation of coating or even three-component wet/solvent spinning can be implemented. This process results in the elimination of certain materials due to their amorphous structure, which leads to increased stiffness (ABS), the use of toxic solvents (TPU)^15^, or other properties that are unsuitable for the desired outcome, such as brittleness (PS). In the case of the OS, the preferred processing methods are braiding and twisting. In theory, any material that is available and producible in fibre or filament could be used. It should be noted, however, that there are certain materials that are not suitable or recommended for use in smart textiles due to their chemical composition. Such materials include PVDF, which contains fluorine atoms. Furthermore, PVC should be eschewed in the context of smart textiles, given the toxic plasticizers inherent to the material. The utilization of PVDF, PTFE and PVC smart textiles on a daily basis result in the release of contaminated microplastics into the environment. In the case of PTFE and PVDF, this phenomenon gives rise to the accumulation of per- and polyfluorinated chemicals (PFAS) in living organisms and the environment. Consequently, humans are consuming an increasing quantity of these carcinogenic substances through the food chain^24,25^. Table 3 provides a summary of the suitability of dielectric materials for CS and OS.

**Table 3:** Summary of suitability of dielectric materials.

	PE	PP	PVC	PU	PET	PTFE	PVDF	PA	ABS	PS	Silicone	TPU
CS	+	+	-	+	-	-	-	+	-	-	+	-
OS	+	+	-	-	+	-	-	+	-	-	+	+

The outer electrode layer, contingent on the system selected, can be constructed from the same materials as the inner electrode. However, for laboratory-scale processes, outer radii of approximately 0.5 mm can be addressed. A pilot plant allows the radius to be reduced to 0.1 mm. Furthermore, the dielectric layer can be nanostructured or fluorinated in order to enhance the glide towards the other filament (SeOS/OS) or the third layer (CS). These processes enhance the inertness of the system and facilitate gliding.

In light of the findings of the theoretical consideration of the systems (i.e., the analysis of the theoretical capacitance and compressive force of the concepts) and the previously discussed facts, the following hypothesis can be posited for the two systems in this publication: Given that both systems utilise a dielectric with comparable permittivity (PET (4.4 F/m) and PU 4.3 F/m), the performance is contingent upon the geometry alone. In the Ba-OS system, a PET multifilament is employed as the dielectric material.

### Proof-of-concept

**Fig. 8:**
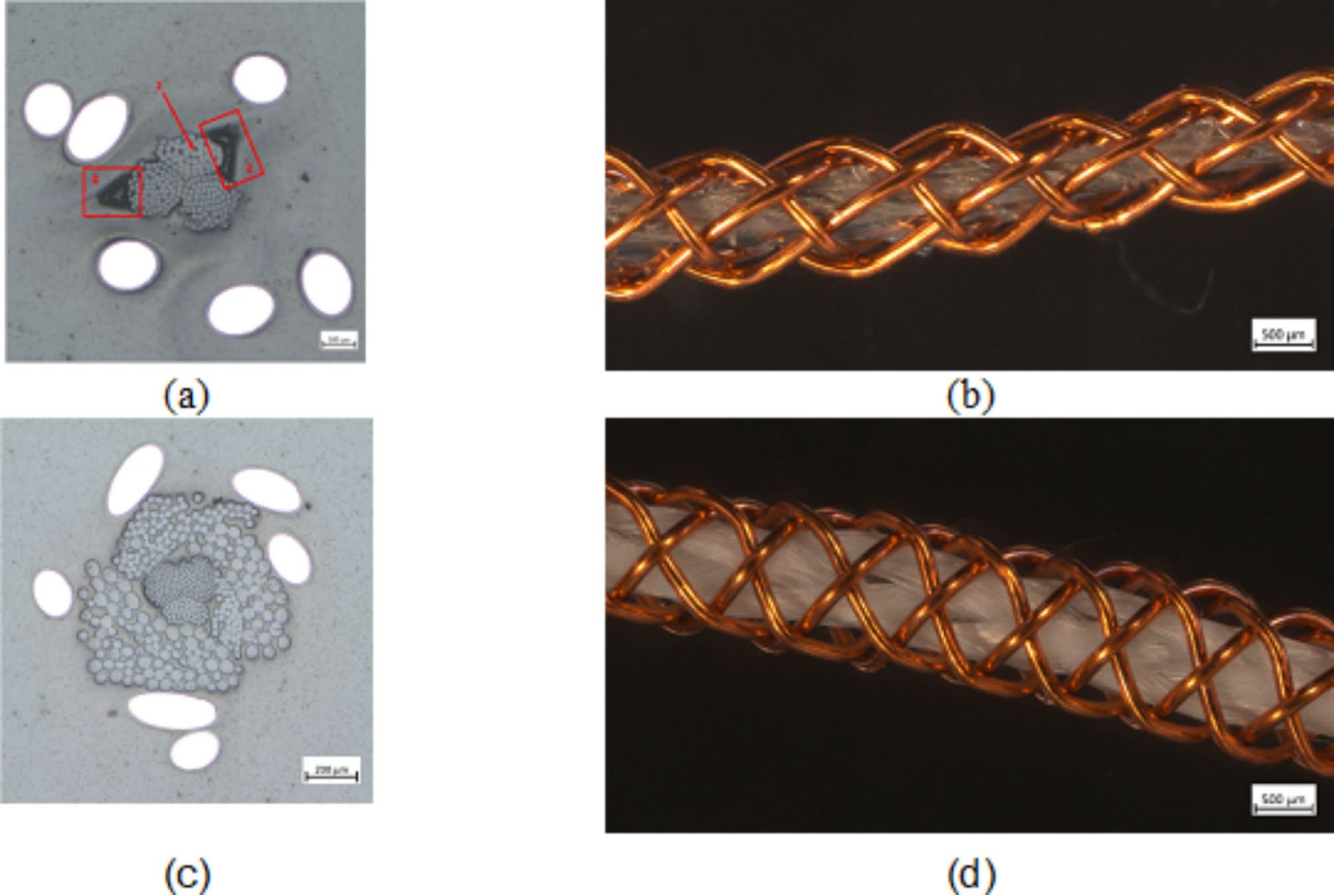
Light microscopy of the cross-section and longitudinal view of the actuator systems for (**a-b**) the PU-SeOS and (**c-d**) the Ba-OS.

The proof of concept was established through the fabrication and testing of PU-SeOS and Ba-OS. Figure 8 illustrates the results of the light microscopy analysis in cross and longitudinal view of the PU-SeOS (a)-(b) and the Ba-OS (c)-(d). The PU coating of the silver-plated yarn in the PU-SeOS is indicated by rectangles and an arrow in Fig. 8 (a). The PU coating is visible between the filaments of the yarn and on the surface of the yarn, though no homogeneous layer has formed.

#### Visual sample inspection

**Fig. 9:**
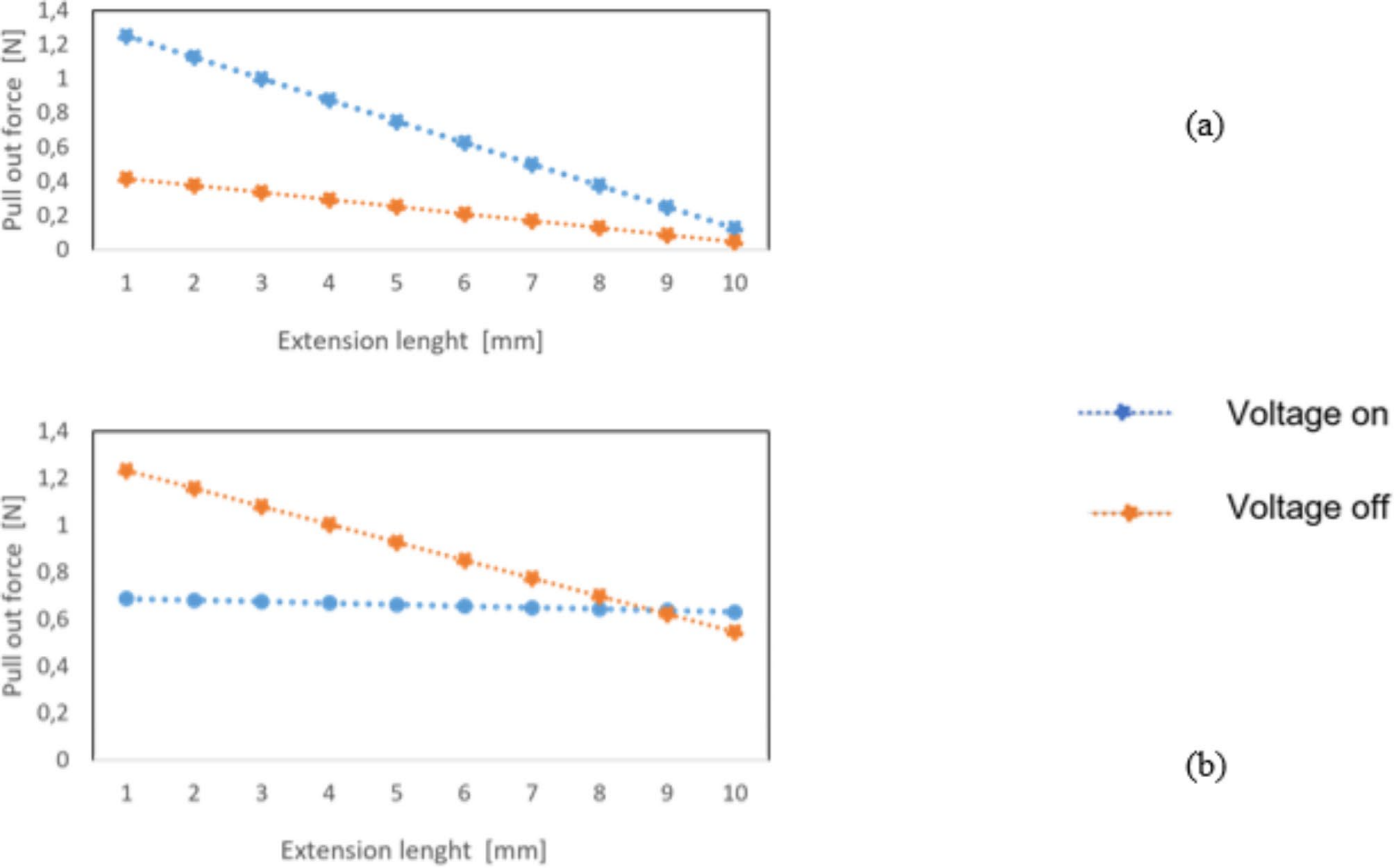
Pullout forces of Ba-OS(a) and PU-SeOS (c) in the state of voltage of (**a**) /on (**b**).

In the longitudinal view (Fig. 8 (b), (d)) the braiding of the mantle is observed to be homogeneous in all systems. The Ba-OS is the largest with a diameter of 1438.66 ± 49.18 μm and the PU-SeOS is the largest with a diameter of 1089.29 ± 61.29 μm (see Fig. 98(b), (d)). The thickness of the dielectric layer is analogous to the size of the actuator systems. The PET layer is the thickest (260.70 ± 101.77 μm) and the PU layer is less thick (78.99 ± 8.044 μm). The size of the systems and the thickness of the layers depend on the choice of materials and the manufacturing method. Given that the core is not entirely round but comprises three filaments, the PU coating is not completely homogeneous around the core. In some areas, the coating has accumulated (see Fig. 9 (a)). Given the relatively elastic nature of PU, the material exhibits partial detachment from the cross-section. This results in the defects appearing larger in the image (Fig. 8 (a)).

The cross-sectional thickness of the adjustable PU layer in the PU-SeOS is less than that of the thicker PET multifilament used in the Ba-OS. In light of the findings presented in Figs. 5 and 6, it can be posited that the actuator performance of the PU-SeOS will be superior. The manufacturing method is also a contributing factor. Given that the dielectric layer in the Ba-OS is comprised of a braid, it can be assumed that the friction when the core is pulled out is higher than in the PU-SeOS, which would consequently impair the actuator performance.

#### Results of the capacitive measurements

Table 4 presents a comparative analysis of the measured capacitance values of the proof-of-concept Ba-OS and PU-SeOS. In contrast, the PU-SeOS exhibited higher resistance values than the Ba-OS. The distinction in the capacitive outcomes between the two systems is attributable to the geometry, given that the two dielectric constant values of the dielectric layer are approximately similar in both systems. As PU-SeOS has a thinner dielectric layer (see Fig. 8), a higher capacitive force was observed in this system. The actual capacitive behaviour of the fibre is represented by the two systems and aligns with the theoretical behaviour previously discussed in the diagrams in Fig. 5 of this section.

**Table 4:** Measured capacitance of Ba-OS and the PU-SeOS.

	Ba-OS	PU-SeOS
Capacitance [pF]	1.74	2.49

#### Results of electrical pull test

Figure 9 illustrates the results of the electrical pullout test of proof-of-concept PU-SeOS (a) and Ba-OS(b).

In the no voltage state, it can be observed that the force decreases with increasing extension length for both systems, as a consequence of the reduction in frictional surface area and mass during core pullout. The PU-SeOS exhibits the highest frictional forces, which can be attributed to the coating properties of the PU coating on the core (Fig. 9 (a)). In contrast, the measured frictional force of the Ba-OS is considerably reduced in the absence of voltage. A comparison of the distribution of the dielectric layers around the core, as shown in Fig. 8, shows that the dielectric layer of Ba-OS is more evenly distributed across the cross-section than that of PU-SeOS. In addition, the PU-SeOS core is directly bonded to the core, whereas the PET filament yarn is only slightly twisted around the core and therefore has less adhesion to the core.

It can be postulated that the capacitance or force will decrease in line with a reduction in the overlapping electrode plates. In the context of the actuator systems, the observed phenomenon is that the compression force decreases as the fibre is pulled out. This behaviour is illustrated in Fig. 9(a), which depicts the voltage-applied results for the Ba-OS. Moreover, in addition to the reduction in force with increasing strain length, the measured forces with applied voltage are higher than those without voltage. In contrast, the compression forces of the PU-SeOS do not demonstrate a reduction in behaviour; rather, they remain constant throughout the entire extension. Given the inhomogeneous nature of the coating and its connection to the braid in certain locations, it can be postulated that the copper threads undergo contraction when the current is applied, thereby exerting additional pressure on the sheath. Furthermore, the longitudinal and transverse views (Fig. 8 (a) and (b)) of the system illustrate the existence of empty space between the core, the dielectric and the sheath. This additional insulating effect, which possesses a high dielectric constant, is exerted by the aforementioned space. An increase in capacitor thickness and additional higher permittivity result in a significant reduction in capacitance. Consequently, a compression force acts on the core, yet no compressive force due to a capacitive effect is observed.

The previous hypothesis regarding the operation of the proof-of-concept actuator systems must be considered invalid in the light of the results obtained. The PU-SeOS, which was identified as a promising variant based on theoretical considerations, showed unsatisfactory results and could not be validated. This is attributed to an inhomogeneous dielectric coating, which results in a higher coefficient of friction of the dielectric layer and therefore a higher frictional force to keep the outer electrode from sliding. Conversely, the Ba-OS showed superior pull-out forces and was validated by the model.

## Conclusion

A comparative analysis of the open system (OS), semi-open system (SeOS) and closed system (CS) concepts reveals a set of advantages, disadvantages and similarities. In conclusion, the CS exhibits superior compression forces in comparison to the SeOS/OS. In general, the capacitance and compression force can be increased by utilising thin dielectric layers and high relative permittivity. An increase in the diameter of the inner electrode results in an enhancement of both capacitance and compression force. In the context of CS, the calculation limits are contingent upon a dielectric layer thickness of 0.1 ∙ 10^− 6^ m, whereas in the case of SeOS/OS, the layer thickness must be at least equivalent to the core diameter. The theoretical forces to be generated are in the N range, although only a fraction of this is required for braking, for example, hand movements. In order to combine the advantages of both concepts, high capacitance and resulting compression forces, the proof-of-concept is implemented in two different actuator systems:

- SeOS with silver-plated yarn as the inner electrode coated with PU dispersion and a braided copper outer electrode. (PU-SeOS)
- OS with silver-plated yarn as the inner electrode with a braided PET layer and a braided copper outer electrode. (Ba-OS)

In addition to the pull-out test, which was conducted to ascertain the proof of concept of all systems, the capacities of the systems were determined. The PU-SeOS exhibits higher capacitance values than the Ba-OS. The difference in the two systems must be geometrical as both systems use dielectric materials with almost the same dielectric constant. It was shown in the microscope images that the PU-SeOS have a thinner dielectric layer. In accordance with the results of the theoretical observation (Fig. 5), the systems with thinner dielectric layers show higher capacitance values. According to the capacitance measurement, the behaviour of the systems’ performances and the theoretical observations are the same.

A validation test was developed for the purpose of evaluating the system. The first phase involved the application of voltage (locked state), while the second phase entailed the removal of the core without voltage (sliding state). In both conditions, the force exhibited a decline as the pull-out distance increased. The Ba-OS exhibited the most favourable sliding and locking force values in both sections of the test. In comparison, the PU-SeOS exhibited the lowest sliding forces and the highest locking forces. Moreover, the behavior of the Ba-OS in response to voltage corresponds to the theoretical considerations, thereby allowing it to be validated. In contrast, the behavior of the PU-SeOS differs from the theoretical expectations, which can be attributed to the inhomogeneity of the dielectric layer.

In this paper, a proof of concept of a fibre-based electrostatic brake has been developed to demonstrate the main functions of a cylindrical shaped brake actuator. However, further work should be carried out in the form of a more systematic approach to evaluate more materials, design and manufacturing processes of the actuator to obtain more detailed information on the behaviour and characteristics.

## Data Availability

The datasets used and/or analysed during the current study are available from the corresponding author on reasonable request. (nadja.schenk@tu-dresden.de)
